# Comparative application of machine learning approaches for body weight prediction in non-descript indigenous goats at different growth stages

**DOI:** 10.14202/vetworld.2025.2878-2887

**Published:** 2025-09-30

**Authors:** Thobela Louis Tyasi

**Affiliations:** Department of Agricultural Economics and Animal Production, University of Limpopo, Sovenga 0727, Limpopo, South Africa

**Keywords:** body weight prediction, classification and regression tree, growth stages, Indigenous goats, machine learning, multivariate adaptive regression splines

## Abstract

**Background and Aim:**

Accurate prediction of body weight (BW) in goats is vital for breeding, feeding, drug administration, and marketing decisions, particularly in resource-limited farming systems where weighing scales are often unavailable. Traditional regression models have been applied but are limited by multicollinearity and non-linearity in body measurement data. This study aimed to evaluate the predictive performance of two machine learning (ML) approaches – Classification and Regression Trees (CART) and Multivariate Adaptive Regression Splines (MARS) – for estimating BW in non-descript indigenous goats across birth, weaning, and yearling stages, compared with stepwise regression models.

**Materials and Methods:**

A total of 100 goats were assessed at three growth stages: Birth (24 h), weaning (4 months), and yearling (12 months). Linear body measurements, body length (BL), sternum height, heart girth (HG), rump height, and withers height, were recorded alongside BW. Correlation analyses, stepwise regression, CART, and MARS models were developed. Model performance was evaluated using the coefficient of determination (R^2^), Pearson’s correlation coefficient (r), Akaike information criterion (AIC), and relative root mean square error (RMSE).

**Results:**

BW showed strong positive correlations with HG and BL across all stages, while associations varied with other morphometric traits. Stepwise regression models exhibited lower predictive power, as indicated by reduced R^²^ values and higher RMSE and AIC scores. In contrast, ML approaches demonstrated superior accuracy. CART consistently outperformed MARS, with R^2^ values of 0.87, 0.94, and 0.99 at birth, weaning, and yearling, respectively. CART also exhibited the highest r values (up to 0.99) and lowest RMSE across training and test datasets.

**Conclusion:**

ML techniques, particularly CART, provide robust and reliable prediction of BW in non-descript indigenous goats, surpassing conventional regression methods. These approaches can guide practical herd management decisions, including optimized feed allocation, drug dosage, and breeding selection, especially in resource-limited settings. While the study underscores CART’s effectiveness, further validation with larger datasets and additional morphometric traits is recommended to enhance generalizability.

## INTRODUCTION

Non-descript indigenous goats are recognized for their strong disease resistance [1] and remarkable adaptability to harsh environmental conditions [2]. Despite these advantages, they generally display slow growth rates and relatively low live body weights (LBW) across different ages [3]. For goat farmers, LBW is a critical parameter used in vaccination, feed management, marketing, and breeding programs aimed at improving the growth performance of subsequent generations [4]. However, many smallholder farmers raising non-descript indigenous goats lack access to weighing scales, making direct measurement of body weight (BW) difficult [5]. Consequently, statistical techniques have been employed to identify key morphometric traits that can serve as reliable predictors of BW.

Conventional regression methods have been widely used for estimating BW in non-descript indigenous goats [6, 7]. Nevertheless, these techniques are limited by their inability to effectively address multicollinearity among predictor variables. In contrast, machine learning (ML) approaches, such as Classification and Regression Trees (CART) and Multivariate Adaptive Regression Splines (MARS), provide more robust alternatives by overcoming multicollinearity and offering greater predictive accuracy [8].

ML has emerged as a powerful tool in animal science, with applications spanning disease prediction, species delimitation, behavioral adaptation, and wildlife monitoring [9]. CART, in particular, is a versatile method capable of handling both classification and regression tasks, with its appeal lying in its simple and interpretable tree-based structure [10]. For example, in Nigerian non-descript goats, CART identified chest girth and neck length as the most influential predictors of BW, explaining 84.2% of the observed variation [11]. MARS, another ML technique, is a non-parametric regression method designed to capture complex, high-dimensional, and non-linear relationships within datasets [4]. It has proven effective in modeling interactions between variables and has been highlighted as a flexible and powerful tool for predicting BW in goats. In Savanna goats, MARS achieved high predictive accuracy (96%) using morphometric traits such as withers height (WH) and heart girth (HG) [4].

According to Mathapo *et al*. [10] and Eyduran [12], ML approaches are invaluable for developing models that identify key traits influencing BW in livestock. Importantly, these approaches offer practical value for resource-limited farmers who may not have access to weighing equipment but still require reliable tools for estimating BW to improve animal management and productivity.

Although studies by Tyasi *et al*. [6] and Tyasi and Putra [7] have explored the use of morphometric traits for predicting BW in goats, most investigations have relied on conventional regression techniques. While useful, these approaches are limited by their inability to adequately account for multicollinearity and non-linear interactions among predictor traits, often resulting in reduced accuracy and generalizability. Recent advances in ML methods, particularly CART and MARS, have shown superior predictive capabilities in livestock BW estimation [8, 11]. However, existing studies are fragmented, with findings varying by breed, age, and region. For instance, CART has been reported to outperform other techniques in Nigerian non-descript goats [11], while MARS demonstrated higher predictive power in Savanna goats [4]. Such inconsistencies suggest that predictive efficiency may be influenced by goat type, growth stage, and the traits considered. Importantly, there remains a paucity of literature on the application and comparative evaluation of CART and MARS for non-descript indigenous goats in South Africa, particularly across critical growth stages (birth, weaning, and yearling). Moreover, no comprehensive studies have benchmarked these ML approaches against traditional regression models in this goat population. Addressing this gap is crucial, given the practical importance of BW estimation for health management, feeding strategies, and selection in smallholder systems where weighing scales are often unavailable.

The present study was therefore designed to investigate the potential of ML approaches for predicting BW in non-descript indigenous goats at different growth stages. Specifically, the study aimed to (i) evaluate the strength of associations between linear body measurements (LBMs) and BW at birth, weaning, and yearling ages; (ii) compare the predictive performance of CART, MARS, and stepwise regression models using goodness-of-fit criteria, including R^2^, root mean square error (RMSE), Akaike information criterion (AIC), and Pearson’s correlation coefficient; and (iii) identify the most influential morphometric traits contributing to BW prediction at each growth stage. By integrating these objectives, the study seeks to provide practical, accurate, and scalable tools for BW estimation that can aid resource-limited farmers in improving herd management, optimizing feed allocation, determining drug dosages, and guiding breeding decisions. Ultimately, this work contributes to strengthening goat production systems by promoting the adoption of innovative ML techniques tailored to indigenous breeds.

## MATERIALS AND METHODS

### Ethical approval

The Animal Research Ethics Committee (AREC) of the University of Limpopo (UL) reviewed and approved the study under approval number AREC/42/2023:UG.

### Study period and location

The study was conducted from June 2023 to May 2024 at the UL Experimental Farm, located 9 km northwest of the UL. The site is characterized by ambient temperature, latitude, longitude, and annual rainfall conditions as previously described by Alabi *et al*. [13].

### Goat management

Management practices followed the protocols outlined by Tyasi *et al*. [6]. Goats were allowed to graze freely during the day and were housed in the afternoon.

### Data collection

A total of 100 non-descript indigenous goats (n = 100) were assessed at three growth stages:

- Birth (24 h after birth)
- Weaning (4 months), and
- Yearling (12 months).

**Figure 1:**
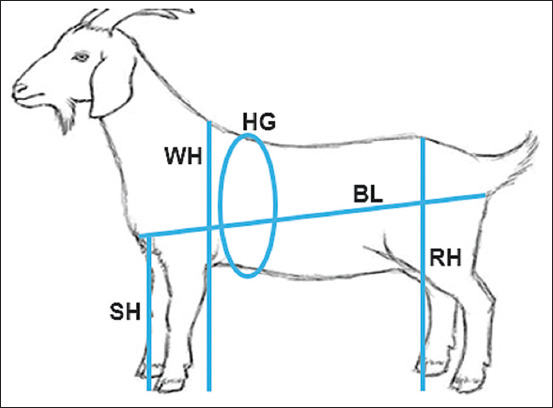
Illustration of linear body measurement traits collected. BL = Body length, SH = Sternum height, HG = Heart girth, RH = Rump height, WH = Withers height.

BW was recorded using a weighing balance scale [14]. LBMs were obtained with a tape measure calibrated in centimeters (cm). The traits measured included body length (BL), sternum height (SH), HG, rump height (RH), and WH (Figure 1). Measurement procedures followed methods described by Norris *et al*. [15] and Lukuyu *et al*. [16].

### Statistical analysis

Data analysis was performed using the Statistical Package for Social Sciences version 29.0 (IBM Corp., NY, USA) [17]. Student’s t-test was used to examine differences across ages for BW and LBM traits. Pearson’s correlation was applied to assess associations among traits.

Two ML methods, CART and MARS, were employed to develop predictive models for BW at birth, weaning, and yearling ages following the procedures of Eyduran *et al*. [18]. Data were split into training (70%) and testing (30%) subsets and validated using 10-fold cross-validation as recommended by Celik and Yilmaz [19]. Goodness-of-fit criteria were applied according to Faraz *et al*. [20].

### Stepwise linear regression analysis

Stepwise regression was conducted to predict BW using only LBM traits that were significantly correlated with BW. The general model equation was expressed as:

Y = a + b1X1 + ⋯bnXn

Where,

Y = Dependent trait (BW at birth, weaning, and yearling)

a = Intercept, b_1_ to b_n_ = coefficient of independent traits, and

X1X1 to Xn = independent traits (LBMs).

Only the correlated LBMs correlated with BW were used for the regression analysis.

### MARS and CART ML approaches

MARS analysis was performed as outlined by Hlokoe *et al*. [21]. The general MARS model was defined as:


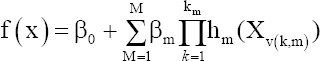


The generalized cross-validation (GCV) error was calculated following the formula explained by Eyduran *et al*. [18].


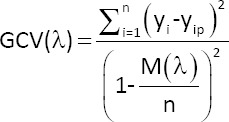


CART analysis was performed according to the methodology of Breiman *et al*. [22].

### Performance evaluation of MARS and CART

The predictive performance of MARS and CART was evaluated using four goodness-of-fit criteria [19]:


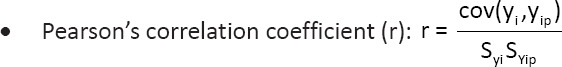



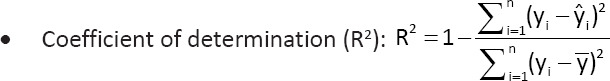



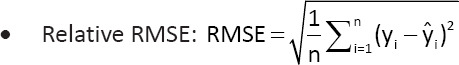



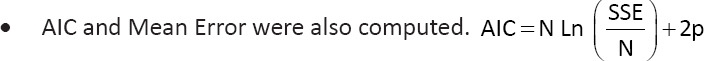


MARS and CART analyses were implemented in R Studio version 4.3.1 using the EhaGof package (Posit PBC) following Eyduran [23].

## RESULTS

### Correlation matrix

The correlation coefficients among BW and LBMs are presented in Table 1, with males represented below the diagonal and females above the diagonal.

**Table 1:** Correlation matrix for males below the diagonal and females above the diagonal.

Traits	BW	HG	RH	BL	WH	SH
Birth						
BW (kg)		0.946**	0.200^ns^	0.879**	0.711**	0.767**
HG (cm)	0.626**		0.378*	0.868**	0.732**	0.723**
RH (cm)	0.521**	0.709**		0.393*	0.634**	0.343*
BL (cm)	0.367*	0.811**	0.424*		0.642**	0.797**
WH (cm)	0.533**	0.880**	0.514**	0.767**		0.757**
SH (cm)	0.507**	0.832**	0.832**	0.798**	0.947**	
Weaning						
BW (kg)		0.317*	0.538**	0.667**	0.134^ns^	0.110^ns^
HG (cm)	0.838**		0.129^ns^	0.171^ns^	0.027^ns^	0.127^ns^
RH (cm)	0.440*	0.224^ns^		0.845**	−0.105^ns^	−0.520**
BL (cm)	0.840**	0.629**	0.751**		−0.048^ns^	−0.363**
WH (cm)	0.287*	−0.018^ns^	0.400*	0.420*		0.524**
SH (cm)	0.461*	0.282*	0.274*	0.310*	0.282*	
Yearling						
BW (kg)		0.533**	0.274*	0.534**	0.501**	0.473*
HG (cm)	0.608**		0.212^ns^	0.510**	0.362*	0.495*
RH (cm)	0.309*	0.195^ns^		0.440*	0.682**	0.544**
BL (cm)	0.788**	0.798**	0.299*		0.425*	0.483*
WH (cm)	−0.101^ns^	0.209^ns^	0.649**	0.235*		0.806**
SH (cm)	0.066^ns^	0.119^ns^	0.820**	0.157^ns^	0.835**	

** = Highly significant at P < 0.01,

* = Significant at P < 0.05, ns = Not significant at P < 0.05, BW = Body weight, SH = Sternum height, HG = Heart girth, BL = Body length, RH = Rump height, WH = Withers height

- At birth, BW showed significant (p < 0.05) positive associations with HG, SH, BL, and WH in both sexes
- At weaning, BW was significantly (p < 0.05) correlated with HG, BL, and RH in both sexes
- At yearling, BW exhibited significant (p < 0.05) correlations with all measured traits in males. In females, however, WH and SH were not statistically associated (p > 0.05) with BW.

### Regression analysis

Stepwise regression outcomes are summarized in Table 2.

**Table 2:** Stepwise linear regression analysis and goodness-of-fit criteria.

Age	Sex	Model	r	R^2^	RMSE	AIC
Birth	Females	BW = −36.96 + 0.91 HG	0.60	0.32	64.32	294.72
		BW = −55.42 + 0.60 HG + 0.61 BL	0.62	0.38	60.45	258.84
		BW = −47.44 + 0.50 HG + 0.51 BL + 0.19 SH.	0.64	0.38	58.76	250.50
		BW = −56.84 + 054 HG + 0.47 BL 0.07 SH + 0.31 WH	0.67	0.44	55.84	237.27
	Males	BW = 28.87 + 0.14 HG	0.33	0.11	194.04	525.22
		BW = −16.66 + 0.01 HG + 0.79 WH	0.40	0.16	192.61	494.49
		BW = −11.11 + 0.01 HG + 0.60 WH + 0.02 RH	0.57	0.33	164.80	401.00
		BW = 36.61–0.04 HG + 4.99 WH + 0.03 RH–3.33 SH	0.78	0.60	103.72	241.07
		BW = −34.05−0.04 HG + 4.87 WH + 0.03 RH−3.27 SH + 0.00 BL	0.92	0.85	43.01	100.19
Weaning	Females	BW = 16.28 0.00 BL	0.19	0.04	6.42	29.40
		BW = 16.51 0.00 BL 0.00 RH	0.22	0.05	6.42	31.06
		BW = 16.68 0.02 BL 0.02 RH + 0.01 HG	0.36	0.13	5.96	30.79
	Males	BW = 15.91 + 0.01 HG	0.28	0.08	10.49	30.28
		BW = 8.32 + 0.00 HG + 0.24 BL	0.51	0.26	8.93	26.73
		BW = 12.55 + 0.30 HG + 0.25 BL 0.44 SH	0.66	0.44	7.19	23.22
		BW = 12.68 + 0.06 HG + 0.22 BL 0.61 SH + 0.43 RH	0.78	0.61	5.33	19.97
		BW = 10.32 + 0.08 HG + 0.21 BL 0.59 SH + 0.38 RH + 0.06 WH.	0.78	0.61	5.68	21.92
Yearling	Females	BW = −29.91 + 0.93 BL	0.53	0.29	68.83	295.92
		BW = −55.42 + 0.61 BL + 0.60 HG	0.62	0.38	60.45	235.90
		BW = −54.10 + 0.46 BL + 0.52 HG + 0.26 WH.	0.66	0.44	55.25	235.90
		BW = −56.84 + 0.47 BL + 0.54 HG + 0.31 WH 0.07 SH	0.67	0.44	55.84	237.27
		BW = −53.08 + 0.56 BL + 0.49 HG + 0.44 WH 0.08 SH 0.24 RH	0.68	0.46	54.60	231.22
	Males	BW = −75.45 + 1.67 BL	0.79	0.63	81.24	221.06
		BW = −72.51 + 1.76 BL 0.12 HG	0.79	0.63	85.74	222.35
		BW = 81.78 + 1.71 BL 0.11 HG + 0.20 RH	0.79	0.63	90.32	222.47

BW = Body weight, SH = Sternum height, HG = Heart girth, BL = Body length, RH = Rump height, WH = Withers height, RMSE = Root mean square error, AIC = Akaike information criterion

- At birth, the best model for females incorporated all measured traits, yielding the highest correlation coefficient (r = 0.67), coefficient of determination (R^2^ = 0.44), and lowest RMSE (55.84) and AIC (237.27). In males, the all-trait model also performed best, with r = 0.92, R^2^ = 0.85, RMSE = 43.01, and AIC = 100.19
- At weaning, the model with all correlated traits in females showed r = 0.36, R^2^ = 0.13, RMSE = 5.96, and AIC = 30.79. In males, the model including HG, BL, SH, and RH provided the best fit with r = 0.78, R^2^ = 0.61, RMSE = 5.33, and AIC = 19.97
- At yearling, females achieved the highest predictive accuracy when all traits were included (r = 0.68, R^2^ = 0.46, RMSE = 54.60, AIC = 231.22). In males, BL alone was the strongest predictor with r = 0.79, R^2^ = 0.63, RMSE = 81.24, and AIC = 221.06.

### MARS ML approach

The MARS models for BW prediction across growth stages are presented in Table 3.

**Table 3:** MARS models.

Age	Model
Birth	BW = 5.37 2.11 * max (0, 38.75 - HG) + 2.78 * max (0, HG - 38.75) 1.78 * max (0, HG - 42.5) + 2.03 * max (0, HG - 43) 0.11 * max (0, 40 - BL) + 1.05 * max (0, BL - 40) + 12.82 * SexM * max (0, 38.75 - HG) 0.06 * max (0, HG - 38.75) * BL + 0.082 * max (0, 38.75 HG SH 0.46 * SexM * max (0, 38.75 - HG) * WH + 0.00 * SexM * max (0, 38.75 - HG) * WH * SH.
Weaning	BW = 5.43 + 0.08 * max (0, 55 - HG) + 0.85 * max (0, 48 - RH) - 2.44 * max (0, RH - 48) + 0.44 * max (0, 45 - BL) + 1.33 * max (0, BL - 45) - 1.79 * max (0, BL - 53) + 0.36 * max (0, 46 - WH) - 0.39 * max (0, 30 - SH) - 0.36 * max (0, SH - 30) - 0.08 * max (0, HG - 55) * max (0, WH - 47) - 0.19 * max (0, HG - 55) * max (0, 47 - WH) - 0.01 * max (0, 57.5 - HG) * max (0, SH - 30) + 0.22 * max (0, HG - 57.5) * max (0, SH - 30) - 0.09 * max (0, 46 - RH) * max (0, BL - 45) + 0.19 * max (0, RH - 46) * max (0, BL - 45) + 0.13 * max (0, 47.5 - WH) * max (0, SH - 30) + 0.03 * max (0, WH - 47.5) * max (0, SH - 30).
Yearling	BW = 47.30 + 0.72 * max (0, RH - 52) + 0.69 * max (0, 59 - RH) -0.92 * max (0, RH - 59) - 3.24 * max (0, RH - 62) - 2.39 * max (0, BL - 62) + 2.17 * max (0, BL - 66) + 291.57 * max (0, BL - 72) - 298.63 * max (0, BL - 72.1) - 1.43 * max (0, 74 - BL) - 0.46 * max (0, 73 - WH) - 0.99 * max (0, WH - 73).

BW = Body weight, BL = Body length, HG = Heart girth, SexM = Males, SH = Sternum height, WH = Withers height, MARS = Multivariate adaptive regression splines

- At birth and weaning, the initial terms of the models included intercepts of 5.37 and 5.43, respectively, with HG serving as a key predictor (cutoff points at 38.75 cm and 55 cm)
- At yearling, the model began with an intercept of 47.30, and RH was identified as the critical predictor with a cutoff point of 52 cm.

### CART ML approach

**Figure 2:**
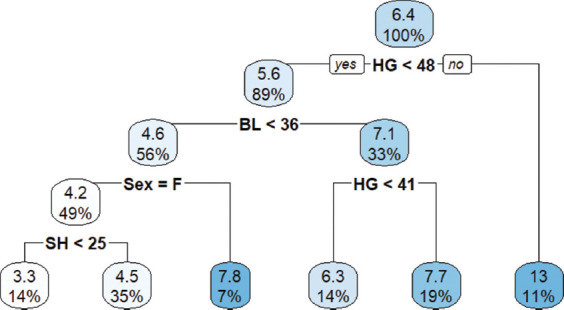
Classification and regression trees model at birth.

**Figure 3:**
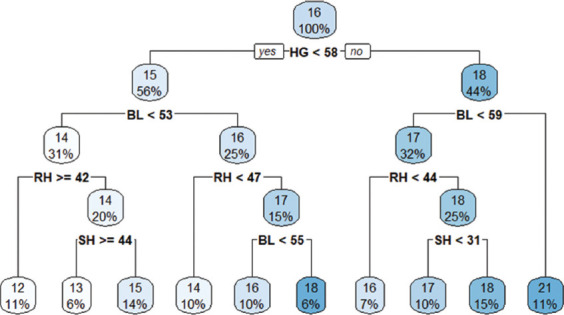
Classification and regression trees model at weaning.

**Figure 4:**
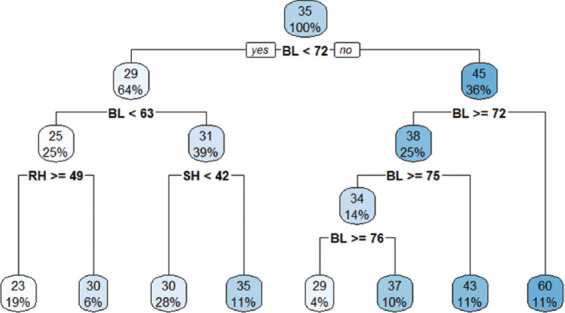
Classification and regression trees model at yearling.

- At birth, the overall mean BW was 6.4 kg. The first CART split showed an average BW of 5.6 kg when BL <36 cm. At the second split, sex (particularly females) influenced BW, with an average of 4.6 kg (Figure 2)
- At weaning, the overall mean BW was 16 kg. The first split showed an average of 15 kg for goats with BL <53 cm. At the second extent of the tree, goats with RH ≥42 cm recorded an average BW of 14 kg (Figure 3)
- At yearling, the overall BW was 35 kg. The first CART split showed an average BW of 29 kg when BL <63 cm. The second split indicated an average BW of 25 kg when RH ≥49 cm (Figure 4).

### Goodness-of-fit criteria

The performance of CART and MARS models was evaluated using correlation coefficient (r), coefficient of determination (R^2^), RMSE, and AIC (Table 4).

**Table 4:** Goodness-of-fit criteria for MARS and CART machine learning approaches.

Criteria	MARS		CART	
	
	Training	Test	Training	Test
Birth				
r	0.10	0.75	0.93	0.93
RMSE	0.27	2.35	1.07	1.22
AIC	−167.05	71.82	9.50	11.25
ME	0.00	−0.49	0.00	−0.54
R^2^	0.99	0.39	0.87	0.83
Weaning				
r	0.99	0.18	0.97	0.94
RMSE	0.12	2.10	0.67	0.97
AIC	−266.73	79.12	−56.94	−1.91
ME	0.00	0.52	0.00	0.25
R^2^	0.99	0.42	0.94	0.88
Yearling				
r	0.99	0.99	0.99	0.99
RMSE	0.70	1.85	0.99	1.34
AIC	−26.88	58.35	−2.23	16.26
ME	0.00	−0.56	0.00	−0.48
R^2^	0.99	0.97	0.99	0.99

ME = Mean error, RMSE = Root mean square error, AIC = Akaike information criterion, CART = Classification and regression trees, MARS = Multivariate adaptive regression splines

- At birth, CART achieved r values of 0.93 (training and testing) with R^2^ of 0.87 and 0.83, outperforming MARS
- At weaning, CART recorded r = 0.97 (training) and 0.94 (testing), with R^2^ values of 0.94 and 0.88, respectively
- At yearling, CART achieved the strongest predictive power with r = 0.99 (training and testing) and R^2^ = 0.99 in both datasets.

Overall, CART consistently outperformed MARS across all growth stages, confirming its robustness in predicting BW in non-descript indigenous goats.

## DISCUSSION

### Importance of LBMs in animal breeding

In animal breeding, identifying LBM traits that influence LBW is crucial for genetic improvement and herd management. According to Rashijane *et al*. [4], LBMs serve as valuable selection criteria in breeding programs to enhance BW in goats. This study applied two ML approaches, MARS and CART, to predict BW in non-descript indigenous goats at birth, weaning, and yearling stages. Accurate BW estimation at these stages is essential for practical management decisions such as feeding, breeding selection, and health interventions.

### Performance of CART versus MARS and regression models

The findings revealed that CART consistently outperformed both MARS and stepwise regression models across all growth stages examined. CART achieved coefficients of determination (R^2^) of 0.87 at birth, 0.94 at weaning, and 0.99 at yearling, demonstrating its strong predictive accuracy. These results are consistent with the ability of CART to model both categorical and continuous variables through decision tree structures, as described by Breiman *et al*. [22].

However, comparisons with other studies revealed varying outcomes. For example, Altay [24] reported that MARS (R^2^ = 0.902) outperformed CART (R^2^ = 0.897) in Honamli goats, likely due to differences in predictor variables used. Similarly, Rashijane *et al*. [4] found MARS to be the best model (R^2^ = 0.959) for Savanna goats aged 2–5 years. These variations highlight the importance of breed type, age, and predictor selection in determining model performance.

### Evidence from related studies

Other studies have reported mixed results when comparing ML algorithms for BW prediction. Haldar *et al*. [9] demonstrated that recursive partitioning and regression tree models outperformed traditional regression methods in Indian Bengal goats. Mokoena *et al*. [5] compared CART, Chi-square Automatic Interaction Detection (CHAID), and exhaustive CHAID in Kalahari Red goats and found CART superior (R^2^ = 0.89). Conversely, Mathapo *et al*. [10] observed CHAID (R^2^ = 0.58) to be more accurate than CART (R^2^ = 0.51) in South African non-descript goats. Beyond goats, Bila *et al*. [8] reported that MARS outperformed CART for BW prediction in Sussex cattle, while Vázquez-Martínez *et al*. [25] found MARS superior in Mexican hair sheep. Collectively, these findings underscore that the relative performance of CART and MARS varies by species, breed, and dataset characteristics.

### Strengths and implications of the present study

A key strength of the present study is its direct comparison of CART, MARS, and regression models for BW prediction at different growth stages in non-descript indigenous goats, a context where such comparisons remain scarce. From a practical perspective, the use of CART provides smallholder and resource-limited farmers with a reliable method of estimating BW without access to weighing scales. This has direct applications in determining feed requirements, drug dosages, and selection decisions for breeding programs, ultimately improving herd management and productivity.

## CONCLUSION

This study evaluated the predictive performance of stepwise regression, MARS, and CART for estimating BW of non-descript indigenous goats at birth, weaning, and yearling stages. The findings demonstrated that BW was strongly correlated with HG and BL across growth stages, with additional traits such as SH, WH, and RH contributing at specific ages. Stepwise regression models yielded relatively low predictive accuracy, with R^2^ values ranging from 0.13 to 0.63, whereas ML approaches provided superior performance. Among these, CART consistently outperformed MARS, achieving R^2^ values of 0.87 at birth, 0.94 at weaning, and 0.99 at yearling, alongside lower RMSE and AIC scores, highlighting its robustness as a predictive tool.

However, the study is limited by the relatively small sample size (n = 100) and the restriction to a single farm population, which may reduce generalizability. In addition, only five LBMs were considered, which may have excluded other important morphometric predictors.

Future studies should expand the sample size across multiple herds and environments, incorporate additional morphometric and genomic traits, and test other advanced ML algorithms such as random forests, gradient boosting, and artificial neural networks. Such approaches could enhance predictive accuracy, support precision livestock farming, and contribute to the establishment of breed standards for indigenous goats.

This study confirms that ML, particularly CART, provides a powerful and practical approach for predicting BW in non-descript indigenous goats. By offering accurate, scalable, and cost-effective alternatives to physical weighing, these models hold promise for advancing sustainable goat production and improving decision-making in resource-limited farming systems.

## DATA AVAILABILITY

The supplementary data can be available from the corresponding author upon a reasonable request.

## AUTHOR’S CONTRIBUTIONS

TLT: Conceptualized and designed the study, performed the study, drafted and revised the manuscript. The author has read and approved the final manuscript.
